# Towards unified and real-time analyses of outbreaks at country-level during pandemics

**DOI:** 10.1016/j.onehlt.2020.100187

**Published:** 2020-10-24

**Authors:** Samuel Soubeyrand, Jacques Demongeot, Lionel Roques

**Affiliations:** a INRAE, BioSP (Biostatistics and Spatial Processes), 84914 Avignon, France; b UGA, Faculty of Medicine of Grenoble, 38700 La Tronche, France

**Keywords:** COVID-19, Data analysis, Epidemiological indicators, Health emergency, Model, Public health, Surveillance data

## Abstract

The management of public health and the preparedness for health emergencies partly rely on the collection and analysis of surveillance data, which become crucial in the context of an emergency such as the pandemic caused by COVID-19. For COVID-19, typically, numerous national and global initiatives have been set up from this perspective. Here, we propose to develop a shared vision of the country-level outbreaks during a pandemic, by enhancing, at the international scale, the foundations of the analysis of surveillance data and by adopting a unified and real-time approach to monitor and forecast the outbreak across time and across the world. This proposal, rolled out as a web platform, should contribute to strengthen epidemiological understanding, sanitary democracy as well as global and local responses to pandemics.

## 1 Introduction

The current COVID-19 pandemic has achieved an unprecedented movement towards the diffusion of information, data and findings, promoted by multiple international initiatives [[1], [2], [3]]. Among information that are relevant to monitor and control COVID-19, real-time indicators describing the on-going epidemic are paramount to rapidly inform decision makers, rally citizens, anticipate risks and assess the impact of containment and mitigation measures. Thus, several open web platforms handling the COVID-19 pandemic with a global vision (i.e., not restrained to specific areas) have emerged and have been used by numerous stakeholders and people across the world to screen and compare outbreaks at country-level and wider scales [[4], [5], [6]]. Essentially, these web platforms provide graphical and dynamic descriptions of raw data, typically counts of confirmed cases and deaths. Here, we promote robust and easily-interpretable indicators grounded on new calculations and indicators offering new insights for developing a unified, real-time and transparent vision of communicable disease epidemics spreading across the world. This proposal could be implemented in a dedicated web platform (such as the prototype that we developed at http://covid19-forecast.biosp.org) and should lead to finer understanding and comparison of epidemic phase during pandemics and, therefore, strengthen sanitary democracy as well as global and local responses to pandemics.

## 2 Designing advanced outbreak indicators

The generalization and globalization of information availability for COVID-19 has facilitated the investigation of differences between countries. Such comparisons can be exploited from a political perspective to support or dispute decision-making. More importantly, they can be used to identify factors favoring or hampering the epidemic at country-level (e.g., those related to population age and social structure, sanitary system, climatic conditions but also lockdown measures) and hence orientate control strategies. They can also be used to alert countries with late outbreak start, but with mortality dynamics following the dynamics of strongly impacted countries that were in the front line of the pandemic. Such country-level comparisons could be attained with a series of real-time indicators routinely calculated and diffused. The indicators we have in mind concern the transmission, the infected population and the mortality, which are key components for the epidemiologist, the decision maker and the citizen to acquire a general understanding of the current and future sanitary situation. We provide thereafter a few examples of indicators that could be obviously enriched for achieving the objectives presented in this note.

## 3 Transmission indicators

**Fig. 1:**
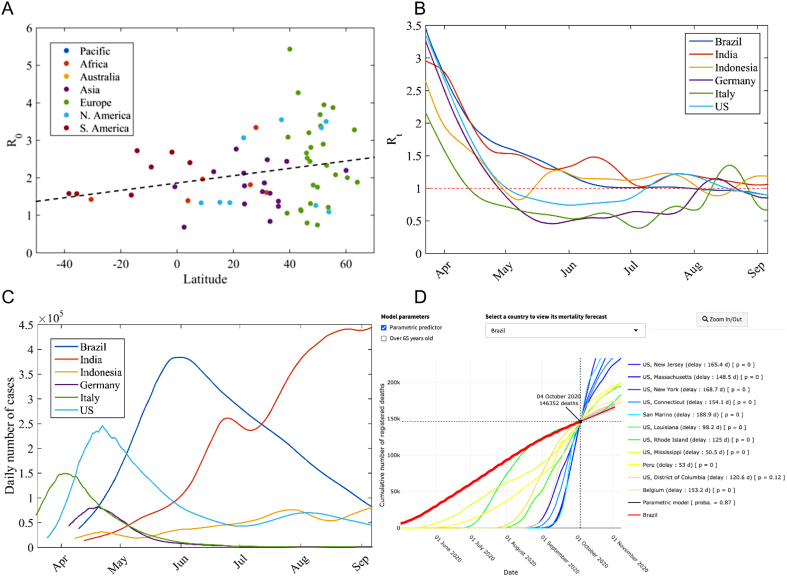
Illustration of advanced indicators for enhancing the standard presentation of outbreak data at the international scale. (A) R0 with respect to latitude (colors of dots depending on the world region). (B) Rt with respect to time, computed from mortality data for three emerging (Brazil, India, Indonesia) and three developed countries (Germany, Italy, the USA). (C) Daily number of cases (confirmed + estimated unreported cases). (D) Mortality trajectory of Brazil up to October 4, 2020, compared to 12 trajectories consisting of 11 real-life dynamics built from data collected in 11 countries/states and 1 parametric dynamic fitted to Brazilian data. Panel D also gives the delay between Brazil and the 12 countries used as benchmarks, the probabilities that Brazil follows each of the benchmark trajectory, as well as a forecast for mortality in Brazil (this forecast being based on the above-mentioned probabilities).

The basic reproduction number (R0) and the effective reproduction number (Rt) of the epidemic form the cornerstone for quantifying the propagation potential of an infectious disease and its evolution across time. However, the way and the frequency at which they are calculated is crucial. The computation of R0 in the initial phase can serve as a benchmark to adequately interpret the variations of Rt in the continuation of the epidemic. However, computing R0 from raw data about confirmed cases leads to unreliable estimates because of a strong dependence on the testing strategy [7]. Instead, one can estimate R0 in a robust and fast way from mortality data using the analytical solution of an approached susceptible-infected-recovered-dead model; see Supplementary Material. This approach allows for easy analysis of correlations with covariates (see Fig. 1, panel A, depicting R0 with respect to latitude) and could be extended to estimate the daily values of R0 along the contagion period. Using the same framework, one can evaluate in real-time the temporal evolution of Rt with a moving observation window, to assess whether the epidemic is still spreading (Rt > 1) or declining (Rt < 1), and to quantify the effect of sanitary measures [8]; see Fig. 1, panel B, which shows striking differences between some emerging and developed countries (as defined by the International Monetary Fund) since April 2020. Details of these computations and the accompanying codes are available as Supplementary Material.

## 4 Infected population indicators

The raw figures of confirmed cases are not satisfactory for comparing the country-level epidemics since the testing strategies are strongly heterogeneous in space, time and age structure. Surveys with adequate sample designs yield more accurate assessment of infected populations, but are today hardly implementable at a high temporal frequency in all countries. Instead, the robust transmission indicator Rt can be plugged in standard epidemiological models (e.g., SIR models and their extensions) to reconstruct the dynamics of the disease accounting for both reported and unreported cases. Thus, one obtains indications in real time about the total infected population (Fig. 1, panel C), including its hidden component that may be relatively large (e.g., for COVID-19 in France, the actual number of infected cases was estimated to be eight times larger than the number of confirmed cases at the start of the epidemic) [9].

## 5 Mortality indicators

Mortality dynamics is typically represented by cumulative curves and daily numbers of deaths, but could be more finely described across time by generalizing simple indicators dedicated to comparisons between countries. A first indicator is given by the delay between a focal country and a benchmark country, where the delay measures for how long the benchmark country has exceeded the current mortality rate in the focal country. The benchmark country is any country that is ahead of time in terms of mortality rate (as defined in [10]) and that is used in a comparative study, because, for instance, it is representative of specific outbreaks (e.g., fast, slow and intermediately rapid outbreaks). This indicator can be easily plotted across time and hence illustrate the relative acceleration or deceleration of the outbreak in the focal country. A second indicator is the probability that a focal country follows the mortality trajectory of a benchmark country. This notion can be extended by considering that the focal country follows a weighted mixture of trajectories of benchmark countries, and this mixture can be monitored across time to evaluate whether the focal country tends to mild or severe mortality dynamics. A third indicator is the forecast over short and medium terms of the mortality. Such a forecast can be derived from the mixture mentioned above [10] (mortality trajectories of ahead-of-time countries playing the role of real-life models) and/or parametric models (either empirical or mechanistic); see Fig. 1, panel D.

## 6 Properties of the indicators

The advanced indicators proposed above should be updated in real time and easily interpretable to provide timely information on the epidemics in all the affected countries. Grounding the indicators on open data and open codes from a transparency perspective should contribute to facilitate their interpretation and their improvement if needed. The indicators should be drawn from unified analyses for enabling benchmarking studies. The calculation of these indicators should be robust and versatile, i.e., adapted to both initial and advanced phases of outbreaks, and achieve a high confidence (in this aim, uncertainty of indicators should be precisely assessed). For large and highly populated countries, the spatial unit used for computing the indicators could be smaller than the country as soon as the epidemic is large enough to account for spatial heterogeneity in terms of epidemic development.

## 7 Developing a shared vision of the pandemic

Our proposal aims at enhancing, at the international scale, the foundations of the analysis of data in pandemic situations by adopting a unified approach to monitor and forecast the outbreak across time and across the world. The series of real-time indicators that we propose to better approach the reality of country-level epidemics underlying raw data could be implemented in a new web platform or enrich those mentioned in the first paragraph of this note. A proof-of-concept has precisely been developed as a Shiny application (available at http://covid19-forecast.biosp.org), which provides the real-time mortality indicators proposed above for many countries, as well as a real-time estimation of the immunity rate across space in France. The platform that we envision would allow a qualitative leap by displaying real-time indicators going beyond the raw figures (typically, numbers of confirmed cases and deaths) provided by existing widespread platforms. This is paramount because, for instance, statistics about confirmed cases generally yield biased country-to-country comparisons due to temporal and spatial heterogeneities in testing strategies (e.g., different numbers of tests per unit of time and unit of population, or different propensities to test at-risk individuals). In contrast, statistics taking into account an estimation of unreported cases for each country, as proposed above, will provide more relevant comparisons. Moreover, designing a web platform facilitating unbiased real-time comparisons between countries would be a crucial tool to rightly alert countries whose sanitary situation evolves towards those of strongly impacted countries. In addition, the platform could incorporate an advanced mode enabling cross-analyses between the sanitary indicators and some country-level variables related to population age distribution, social structures, health system, economics, climate and control strategy. By enabling such cross-analyses, the platform would offer an exploratory tool to roughly evaluate, for instance, the effect of lockdown measures, and to establish hypotheses to be tested in further studies (namely mechanistic hypotheses [11], but also hypotheses addressing questions about data quality). The dissemination of the platform towards health institutions, the media, the public and private stakeholders should contribute to reinforce relevancy of governmental decisions, sanitary democracy and health communication intended to educate people [12].

To further improve our vision of pandemics, the indicators proposed above, which are primary keys for the management of health emergencies caused by COVID-19-like diseases, could be supplemented by information on data quality and genetic signals. Furthermore, in the case of zoonoses, the one-health perspective could be better taken into account by considering surveillance data collected from wild animal populations in parallel with surveillance data collected from human populations.

At the pandemic horizon (before the availability of efficient and consensual vaccination or therapy) for COVID-19-like crises, the unified and real-time analyses that we propose could help in better monitoring spatio-temporal variations of disease propagation and impact, alerting on critical situations, and comparing efficiency of containment and mitigation measures. At the post-pandemic horizon, they could further help in assessing and comparing the beneficial impact of vaccination and therapy at global scale, and pointing out regions requiring attention (using a homogeneous and expectedly unbiased prism). Setting up the advanced platform we mentioned for the current COVID-19 pandemic is definitely challenging, but this pandemic must be used to conceive and develop it.

## Funding

None.

## Data availability

Data are publicly available at https://github.com/CSSEGISandData/COVID-19/ and https://covidtracking.com/data.

## Declaration of Competing Interest

Authors declare no competing interests.

## Appendix A Supplementary data

Supplementary material Image 1
