# Supplementary material for: Towards unified and real-time analyses of outbreaks at country-level during pandemics
Source: One Health. 2020 Oct 24;11:100187. doi: 10.1016/j.onehlt.2020.100187 (PMC7584491; doi:10.1016/j.onehlt.2020.100187)
Supplement: Supplementary file 1 — Supplementary material [file mmc1.pdf]

Towards unified and real-time analyses of outbreaks at country-level during pandemics

—–

Supplementary Material

Samuel Soubeyrand[1], Jacques Demongeot[2], Lionel Roques[1]

[1] INRAE, BioSP (Biostatistics and Spatial Processes), 84914 Avignon, France
[2] UGA, Faculty of Medicine of Grenoble, 38700 La Tronche, France

Transmission and infected population indicators

Standard mechanistic epidemiological models rely on SIR (Susceptible-Infected-Removed) systems of ordinary differential equations and their extensions (for examples of application to the COVID-19 epidemic, see [5, 6]). Assume here that the dynamics of the epidemic are described by the following SIRD compartmental model:

$$\begin{cases} S'(t) = -\dfrac{\alpha(t)}{N}\, S(t)\, I(t), \\ I'(t) = \dfrac{\alpha(t)}{N}\, S(t)\, I(t) - (\beta+\gamma)\, I(t), \\ R'(t) = \beta\, I(t), \\ D'(t) = \gamma\, I(t), \end{cases} \tag{1}$$

with S the susceptible population, I the infectious population, R the recovered population, D the number of deaths due to the epidemic and N the total population (which is assumed to be constant, thereby neglecting the effect of death on the total population size). The time-dependent coefficient $\alpha(t)$ is the contact rate and $1/\beta$ is the mean time until an infectious becomes recovered. The results in [1] indicate that infectiousness starts 2 to 3 days before symptom onset and declines significantly 8 days after symptom onset. Based on these observations we assume here that the mean duration of the infectiousness period is $1/\beta = 10$ days. In [2], the duration of the incubation period was estimated to have a mean of 5.2 days. Thus, the mean duration of the non-infectious exposed period is relatively short (about 2 to 3 days), and can be neglected without much differences on the results, as shown in [3]. The parameter γ corresponds to the death rate of the infectious, which has been estimated in [5, 6], based on French data: $\gamma = 8 \cdot 10^{-4}$ (corresponding to an infection fatality rate of $\gamma/(\gamma+\beta) \approx 0.8\%$).

Computation of R_0 based on the number of observed deaths. To compute the basic reproduction number, we assume that the contact rate α is constant at the beginning of the epidemic, during a period (t_0, t_1). The date t_0 is defined as the first day such that the observed number of deaths $\hat{D}_{t_0}$ exceeds 10, and t_1 is the first date where the number of deaths $\hat{D}_{t_1}$ crosses the value 100. The basic reproduction number can be computed as [4]:

$$R_0 = \frac{\alpha}{\beta+\gamma}.$$

As $S \approx N$ at the early stage of the epidemics and since $\beta+\gamma \approx \beta$, we have:

$$I'(t) = I\left(\alpha\,\frac{S}{N} - (\beta+\gamma)\right) \approx I\,\beta\,(R_0 - 1),$$

which can be solved explicitly, leading to

$$\begin{cases} I(t) = I_0\, e^{\beta\,(R_0-1)\,(t-t_0)}, \\ R(t) = \dfrac{I_0}{R_0-1}\left(e^{\beta\,(R_0-1)\,(t-t_0)} - 1\right), \\ D(t) = \hat{D}(t_0) + \dfrac{\gamma}{\beta}\dfrac{I_0}{R_0-1}\left(e^{\beta\,(R_0-1)\,(t-t_0)} - 1\right), \end{cases}$$

with $R(t_0) = 0$, $I(t_0) = I_0$ and $D(t_0) = \hat{D}_{t_0}$.

Fixing the value $\hat{D}_{t_1}$ (cumulated number of recorded deaths on day t_1), we get:

$$I_0 = \frac{\beta}{\gamma} \frac{(R_0 - 1)(\hat{D}_{t_1} - \hat{D}_{t_0})}{e^{\beta\,(R_0-1)\,(t_1-t_0)} - 1}.$$

Finally,

$$D_t = \hat{D}_{t_0} + (\hat{D}_{t_1} - \hat{D}_{t_0}) \frac{e^{\beta\,(R_0-1)\,(t-t_0)} - 1}{e^{\beta\,(R_0-1)\,(t_1-t_0)} - 1}, \qquad (2)$$

for all $t \in (t_0, t_1)$. R_0 is then computed by fitting the above formula to the data $\hat{D}_t$ with a standard nonlinear curve fitting procedure (using Matlab® Curve Fitting Toolbox®), over the period (t_0, t_1).

Computation of R_t based on the number of observed deaths. The effective reproduction number is defined as:

$$R_t = \frac{\alpha}{\beta + \gamma} \frac{S(t)}{N}.$$

The value of R_t is computed with the same procedure as R_0 but instead of fitting (2) over a fixed period (t_0, t_1), we apply the fitting procedure over a dynamical time period $(t - \tau, t)$ (we use the same approximation as above $\beta + \gamma \approx \beta$ and we assume that S/N is approximately constant over the considered period of time). Thus, R_t is computed by fitting to the data $\hat{D}_s$ for $s \in (t - \tau, t)$ (same fitting procedure as above) the following formula:

$$D_s = \hat{D}_{t-\tau} + (\hat{D}_t - \hat{D}_{t-\tau}) \frac{e^{\beta\,(R_t-1)\,(s-(t-\tau))} - 1}{e^{\beta\,\tau\,(R_t-1)} - 1}. \qquad (3)$$

In the illustrations presented in the main text, the size of the window was $\tau = 28$ days.

Computation of the actual number of cases. We use the above procedure to compute R_t, and to deduce $\alpha(t) \approx \beta\, R_t$. The computation of the number of cases begins at some time t_0 defined again as the first day such that the observed number of deaths $\hat{D}_{t_0}$ exceeds 10. To initialise the number of infectious $I_0 = I(t_0)$, we use the equation $D'(t) = \gamma\, I(t)$, and we define I_0 as $1/\gamma \times$ (mean number of deaths over the period ranging from 5 days before t_0 to 5 days after t_0). $S(t_0) = N$ corresponds to the population size in the considered country.

Then, the solution of the system (1) can be computed with a standard numerical algorithm, using e.g. Matlab® *ode45* solver. The daily number of cases is computed as $S(t-1) - S(t)$, the variation in the number of susceptibles between two consecutive days.

Computer codes

Computer codes implementing the calculations detailed above were developed with Matlab®. Computer codes for mortality indicators were developed with the `R` statistical software. The former codes were used to produce Panels A-C in Figure 1 in the main text, and the latter codes were used to generate Panel D. All these codes are available at `https://doi.org/10.5281/zenodo.4071893`.

References

[1] Xi He, Eric HY Lau, Peng Wu, Xilong Deng, Jian Wang, Xinxin Hao, Yiu Chung Lau, Jessica Y Wong, Yujuan Guan, Xinghua Tan, et al. Temporal dynamics in viral shedding and transmissibility of COVID-19. *Nature Medicine*, pages 1–4, August 2020.

[2] Qun Li, Xuhua Guan, Peng Wu, Xiaoye Wang, Lei Zhou, Yeqing Tong, Ruiqi Ren, Kathy SM Leung, Eric HY Lau, Jessica Y Wong, et al. Early transmission dynamics in Wuhan, China, of novel coronavirus–infected pneumonia. *New England Journal of Medicine*, 382:1199–1207, 2020.

[3] Z Liu, P Magal, Ousmane Seydi, and Glenn Webb. A COVID-19 epidemic model with latency period. *Infectious Disease Modelling*, 2020.

[4] J D Murray. *Mathematical Biology.* Third edition, Interdisciplinary Applied Mathematics 17, Springer-Verlag, New York, 2002.

[5] Lionel Roques, Etienne Klein, Julien Papaix, Antoine Sar, and Samuel Soubeyrand. Using early data to estimate the actual infection fatality ratio from COVID-19 in France. *MDPI Biology*, 9(5):97, 2020.

[6] Lionel Roques, Etienne K Klein, Julien Papaix, Antoine Sar, and Samuel Soubeyrand. Impact of lockdown on the epidemic dynamics of COVID-19 in France. *Frontiers in Medicine*, 2020, DOI: 10.3389/fmed.2020.00274.
